# Factors influencing parental COVID-19 vaccination willingness for children in Japan

**DOI:** 10.1016/j.jvacx.2024.100528

**Published:** 2024-07-18

**Authors:** Mami Ueta, Alton Cao, Michio Murakami, Hana Tomoi, Stuart Gilmour, Keiko Maruyama-Sakurai, Yoshihiro Takayama, Yoshitake Takebayashi, Masahiro Hashizume, Rauniyar Santosh Kumar, Hiroyuki Kunishima, Wataru Naito, Tetsuo Yasutaka, Satoshi Kaneko, Hiroaki Miyata, Shuhei Nomura

**Affiliations:** a Department of Global Health Policy, Graduate School of Medicine, The University of Tokyo, Tokyo, Japan; b Department of Health Policy and Management, School of Medicine, Keio University, Tokyo, Japan; c Center for Infectious Disease Education and Research, Osaka University, Osaka, Japan; d Department of Infectious Disease Epidemiology, Faculty of Epidemiology and Population Health, London School of Hygiene and Tropical Medicine, London, UK; e School of Tropical Medicine and Global Health, Nagasaki University, Nagasaki, Japan; f Graduate School of Public Health, St. Luke’s International University, Tokyo, Japan; g Department of International Health and Medical Anthropology, Institute of Tropical Medicine, Nagasaki University, Nagasaki, Japan; h Division of Infectious Diseases, Okinawa Prefectural Chubu Hospital, Okinawa, Japan; i Department of Health Risk Communication, Fukushima Medical University School of Medicine, Fukushima, Japan; j Ocean Policy Research Institute, Sasakawa Peace Foundation, Tokyo, Japan; k Department of Infectious Diseases, St. Marianna University School of Medicine, Kanagawa, Japan; l Research Institute of Science for Safety and Sustainability, National Institute of Advanced Industrial Science and Technology (AIST), Ibaraki, Japan; m Research Institute for Geo-Resources and Environment, National Institute of Advanced Industrial Science and Technology (AIST), Ibaraki, Japan; n Department of Ecoepidemiology, Institute of Tropical Medicine, Nagasaki University, Nagasaki, Japan; o Tokyo Foundation for Policy Research, Tokyo, Japan

**Keywords:** COVID-19, Japan, Child, Vaccination, Willingness, Parent

## Abstract

**Objectives:**

This study aims to investigate the factors influencing parental willingness in COVID-19 vaccination for children in Japan in light of the introduction of pediatric vaccines.

**Methods:**

An online survey was conducted in February 2022, coinciding with the imminent start of pediatric COVID-19 vaccinations in Japan. It assessed attitudes toward vaccine uptake and included questions about health-related attributes, psychological considerations, and sources of COVID-19 information. Data from 2,419 respondents who had children under the age of 12 were analyzed using multinomial logistic regression to identify factors associated with parental willingness towards COVID-19 vaccination for children. The outcomes were “agree” (in favor of vaccination), “not sure” (undecided), with “disagree” (against vaccination) as the reference category.

**Results:**

Among participants supportive of vaccination (“agree” compared to the “disagree” reference), salient determinants included: gender (Men in reference to women: odds ratio [OR] 1.54; 95 % confidence interval [CI] 1.11–2.15), highest educational level (Junior College/Vocational in reference to under high school: OR 0.61; 95 % CI 0.40–0.93, Bachelor’s /Master’s/Doctoral degree in reference to under high school: OR 0.59; 95 % CI 0.42–0.84), perception of benefits of COVID-19 vaccination (Significant in reference to Insignificant: OR 2.04; 95 % CI 1.26–3.28), perception of risks of COVID-19 vaccination (Significant in reference to Insignificant: OR 0.28; 95 % CI 0.19–0.42, Neutral in reference to Insignificant: OR 0.48; 95 % CI 0.33–0.71), the number of referenced information sources utilized for COVID-19 was associated with attitudes towards children’s vaccination (OR 1.02; 95 % CI 1.00–1.04).

**Conclusion:**

The study highlights the multifaceted factors influencing parents’ COVID-19 vaccination attitudes for their children, encompassing socioeconomic, health, psychological, and informational aspects. Factors like cautious information gathering, vaccine concerns and diverse referenced information sources impact willingness. To facilitate informed decision-making, essential measures include government risk communication, widespread vaccine information dissemination, and enhancing parents’ health information accessibility and evaluation skills are important.

## 1 Introduction

In the context of pediatric immunization against COVID-19 in Japan, initially, in response to the rising number of COVID-19 cases and the emergence of severe symptoms in children, the Ministry of Health, Labour and Welfare granted pharmaceutical approval for COVID-19 vaccines for children aged 5–11 on January 21, 2022. Subsequently, on February 21, 2022, it was designated as a legal vaccination [1], [2]. In September 2022, the Japanese government officially commenced recommending COVID-19 vaccination for children aged 5 and above. In October 2022, the government extended the approval for pharmaceutical use and legal vaccination to children aged 6 months and older, thereby expanding the scope of recommendation [1], [2]. Additionally, the Japan Pediatric Society highlighted the importance of COVID-19 vaccination for children to protect child health and mitigate the impact of the ongoing pandemic [1], [2], [3], [4].

In spite of these official recommendations, only 24.5 % of children between the ages of 5 and 11, and 4.3 % of children between the ages of 6 months and 4 have been inoculated with the first dose of the COVID-19 vaccine in Japan as of November 2023 [5]. The successful COVID-19 vaccination program for children requires parental acceptance of vaccination. However, previous research conducted in Japan as of April 2021 revealed that parents had relatively negative sentiments towards COVID-19 vaccines: 42.7 % of 1,100 participants were not sure if they got their children vaccinated against COVID-19 and 14.4 % were unwilling to do so [6]. It is essential to understand the underlying factors influencing parents' willingness towards COVID-19 vaccination for children. This understanding is key to developing a tailored strategy to increase the parental likelihood of accepting COVID-19 vaccines for children [3], [7].

Some studies have investigated factors associated with parents' willingness for COVID-19 vaccination for children. The sociodemographic attributes of parents, including female gender, younger age and lower education level have been found to be associated with vaccination hesitancy for their children [8]. Psychographic attributes such as parents’ fear of side effects and uncertainty about vaccine safety and effectiveness were also found to be related with pediatric vaccination hesitancy [6]. Furthermore, parents who trusted social media over official information sources as their primary source of COVID-19 information were found to be more hesitant towards pediatric vaccination [8]. Additionally, previous studies indicate that providing knowledge by healthcare providers is crucial in reducing decisions to decline vaccines [9].

However, since most existing literature on pediatric vaccine hesitancy in Japan was conducted before pharmaceutical approval, parental willingness to vaccinate children was only hypothetical. There is a knowledge gap regarding parental perspectives in the context where COVID-19 vaccines for children became available and more feasible. This study aims to estimate parental willingness for COVID-19 vaccination in Japan and its underlying factors based on recent survey results. By understanding parents' motivations for or against vaccination, this study can help policymakers and communities develop targeted strategies to increase pediatric COVID-19 vaccine uptake and improve preparedness for future pandemics.

In this study, we analyzed a nationwide online survey conducted in February 2022, focusing on parental willingness to vaccinate children against COVID-19. The survey followed the January 21, 2022, pharmaceutical approval of the COVID-19 vaccine for children aged 5–11 in Japan, just before vaccinations began. This timing offers a unique opportunity to explore factors influencing parental decision-making, as information on childhood vaccination was widely available and parents were actively considering vaccination for their children.

## 2 Materials and methods

### 2.1 Data source

This study analyzed data from a longitudinal series of surveys conducted in 2021 and 2022. Briefly, these surveys were conducted online in Japanese through a web-based research company (Cross Marketing, Inc.), with participants aged 20 and older [10]. This company had approximately 7.67 million panel members through voluntary registration as of 2023. Responses were collected online and initially stored on the servers of research company. Upon closure of each survey, the data was compiled and securely transferred to us in CSV format.

The questionnaire used in the online survey was peer-reviewed by experts from the Japan Epidemiological Association, the Japanese Society of Infectious Diseases, and the COVID-19 Information Value Improvement and Link project (CIVIL project) based on a thorough review of previous studies [11], [12].

The 2021 survey was conducted between 26 February and 4 March 2021, which recruited approximately 30,000 people through a quota sampling method based on age (at the time of the survey), gender, and prefecture attributes matched data from the 2015 National Census to ensure that the sample was representative of the population [13]. Recruitment in the 2021 survey was on a first-come, first-served basis, and was closed when the number of participants in the above criteria was reached.

The 2022 survey was conducted as a follow-up to the survey in 2021[14], [15], [16], [17]. It was conducted between 4 February 2022 and 24 February 2022 with 19,195 respondents (response rate 63.9 %), using the same quota sampling method as the 2021 survey.

This study analyzed data of 2,419 of respondents who participated both 2021 and 2022 surveys and the second survey who answered that they had children under the age of 12, utilizing information from both the first and second surveys.

The detailed procedures of the survey are as follows. Respondents had to provide their informed consent before they proceeded to the questionnaire response page. They were informed about the use of data for research purposes and the anonymization of their responses prior to submission. This information was clearly presented on the consent page of the survey. No reminders were sent to the respondents. The survey system was designed to prevent a single individual from responding multiple times.

The survey’s participants were awarded points that could be used for items from partner companies for online shopping by answering all questions in the survey, so there was no missing data in these surveys. Respondents who selected “other” for gender were excluded due to the small number of respondents.

### 2.2 Data analysis

In this study, we primarily used explanatory variables from the most recent second 2022 survey. All questions were closed-ended and in a single- or multiple-response format, including “yes/no” scales, ordinal scales, nominal scales, and Likert scales. Unless otherwise noted, all responses provided were the latest at the time of the survey. Further information on the questionnaire development is described in other papers [15], [17]. The original text of all the questions used in this statistical model, along with their English translations, are provided in Supplementary Table 1.

### 2.3 Outcome measures

Parental willingness to vaccinate their children against COVID-19 was measured by asking, “When it becomes possible for children under 12 to receive the COVID-19 vaccine in the future, which of the following applies to you?”. The response options were “agree (have children of the relevant age) ”, “agree (have no children of the relevant age)”, “disagree (have children of the relevant age)”, “disagree (have no children of the relevant age)”, “not sure (have children of the relevant age)”, and “not sure (have no children of the relevant age)”. Given the primary target population in this study, those having no child were excluded from the analysis. Thus, those who answered ”agree (have children of the relevant age)“, ”disagree (have children of the relevant age)“ and ”not sure (have children of the relevant age)” are included. Hereafter, these are referred to as the groups, “agree”, “disagree”, and “not sure”.

### 2.4 Other variables

The variables considered in this study were selected through a backward stepwise variable selection method to narrow down a comprehensive set of survey questions that might have associations with parental willingness regarding children's COVID-19 vaccination. These variables can be broadly divided into four categories: demographic and socioeconomic variables, health-related variables, psychological variables, and variables related to information sources regarding COVID-19.

Regarding demographic and socioeconomic variables, region of residence and the areas which were subject to intensive measures for preventing the spread of COVID-19 infection during the survey were included. The areas subject to intensive measures are specific regions designated by the government when there is a high risk of the spread of COVID-19 infections and a potential for disruption to the healthcare system [18], [19]. In addition to these variables, gender, age, educational achievement, occupation, and household income brackets in 2020 are also included. With regards to health-related variables, the respondents were asked about the status of their routine vaccinations, as well as their personal COVID-19 vaccination behavior and of those around them.

Psychological variables include perception of benefits and disadvantages of COVID-19 vaccination, degree of trust in public authorities, perspectives on the influence of peer vaccination on one's own vaccination, the degree of anxiety for COVID-19, and the disruption to their daily life by COVID-19. Regarding sources of information about COVID-19, five categories of variables were selected to evaluate the level of trust in information sources, including healthcare specialists such as doctors; mass media, social media and websites; government agencies; close friends and family members. The degree of trust was rated on a scale of 1 (not at all trustworthy) to 4 (very trustworthy). In addition, the total number of referenced information resources for COVID-19 vaccination was included as a variable. The respondents provided their answers regarding the number of information resources they utilized out of the provided 30 options. The trust values regarding information sources were treated as continuous variables, and means and standard deviations [20] were calculated.

### 2.5 Statistical analysis methods

In this study, we analyzed the outcome related to children's vaccination using a multinomial logistic regression model, with “disagree” coded as 0, and “agree” and “not sure” coded as 1. To explore factors related to the outcome, willingness to vaccinate children against COVID-19, the selection of explanatory variables were firstly selected from a wide range of survey questions that could be related to, and refined through a backward stepwise variable selection method removing variables with p ≥ 0.2 and adding terms with p < 0.05 [21]. Further information on the explanatory variables actually used in the last model by this procedure is mentioned in the “other variables” section.

To address concerns related to variable overfitting and multicollinearity, adjustments were made to several variable options to ensure that correlation remained below 0.7. For instance, for levels of trust in information sources, the initial pool of 30 information source choices was consolidated into 5 broader categories. Similarly, in pursuit of enhanced analytical clarity, certain response options were aggregated. Also, to facilitate a better understanding of respondents' inclinations, the response choices in surveys were standardized by consolidating options. Moreover, when dealing with response options exceeding three and falling below 200 respondents, attempts were undertaken to amalgamate these categories within the other categories, whenever it was feasible. Each category of “information sources”, which were calculated as the average of multiple items in the questionnaire, was assessed for reliability through the Cronbach's alpha test or Spearman-Brown test. For detailed descriptions of the amalgamated categories, refer to Supplementary Table 2.

Odds ratios and 95 % confidence intervals for the results were estimated by multinomial logistic regression and were considered statistically significant if two-sided p < 0.05. All analyses were performed using STATA/BE version 17.0.

## 3 Results

### 3.1 Outcome distribution

The response rate for the second survey was 63.9 %, with 19,195 respondents participating. Among a total of 2,419 parents with children of eligible age, 468 individuals (19.4 %) responded as “disagreed”, 1,049 individuals (43.4 %) responded as “agreed”, and 902 individuals (37.3 %) responded as “not sure”.

### 3.2 Sociodemographic characteristics

The characteristics of participants is shown in Table 1 and Supplementary Table 3. Most participants (34.9 % of 2419 participants) were from the Kanto region and the proportion of individuals residing in the areas subject to intensive measures for preventing the spread of COVID-19 infection during the survey period amounted to 90.7 % of the total population. About half of the participants (53.2 %) were male. The average age of participants was 44.4 years (SD 13.1 years). More than 50 % of participants (54.0 %) had a bachelor's degree or higher. The most common occupations were the category of student and homemaker (21.7 %) and manufacturing (16.3 %). The most common income bracket was in the range of less than 3 million yen (15.1 %).

**Table 1:** **Characteristics of Parents.** (*Extracting only the major characteristics (Please refer to Supplementary Table 3 for all characteristics)).

	Disagree (n = 468, 19.4 %)	Agree (n = 1049, 43.4 %)	Not sure (n = 902, 37.3 %)	Total (n = 2,419)
***Sociodemographic characteristics***				
**Gender**				
Women	256 (22.6/54.7)	392 (34.6/37.4)	485 (42.8/53.8)	1133 (100/46.8)
Men	212 (16.5/45.3)	657 (51.1/62.6)	417 (32.4/46.2)	1286 (100/53.2)
Total	468 (19.4/100)	1049 (43.4/100)	902 (37.3/100)	2419 (100/100)

**Highest educational level**				
Middle/High school	114 (16.9/24.4)	286 (42.3/27.3)	276 (40.8/30.6)	676 (100/28.0)
Junior College/Vocational	93 (21.2/19.9)	165 (37.7/15.7)	180 (41.1/20.0)	438 (100/18.1)
Bachelor's/Master's /Doctoral	261 (20.0/55.8)	598 (45.8/57.0)	446 (34.2/49.5)	1305 (100/54.0)
Total	468 (19.4/100)	1049 (43.4/100)	902 (37.3/100)	2419 (100/100)

**Occupation**				
Medical and Healthcare workers	41 (19.3/8.8)	89 (41.8/8.5)	83 (39.0/9.2)	213 (100/8.8)
Manufacturing	69 (17.5/14.7)	193 (49.0/18.4)	132 (33.5/14.6)	394 (100/16.3)
Student/Homemaker	128 (24.3/27.4)	161 (30.6/15.4)	237 (45. 2/ 16.3)	526 (100/21.7)
Other	230 (17.9/49.2)	606 (47.1/57.8)	450 (35.0/49.9)	1286 (100/53.2)
Total	468 (19.4/100)	1049 (43.4/100)	902 (37.3/100)	2419 (100/100)

***Health-related characteristics***				
**Routine vaccinations**				
Yes	158 (19.9/33.8)	364 (45.9/34.7)	271 (34.2/30.0)	793 (100/32.8)
Some	76 (17.7/16.2)	223 (51.9/21.3)	131 (30.5/14.5)	430 (100/17.8)
No	160 (20.8/34.2)	319 (41.5/30.4)	290 (37.7/32.2)	769 (100/31.8)
Not sure	74 (17.3/15.8)	143 (33.5/13.6)	210 (49.2/23.3)	427 (100/17.7)
Total	468 (19.4/100)	1049 (43.4/100)	902 (37.3/100)	2419 (100/100)

**COVID-19 vaccination status**				
No	172 (51.2/36.8)	44 (13.1/4.2)	120 (35.7/13.3)	336 (100/13.9)
Yes or Will	296 (14.2/63.3)	1005 (48.3/95.8)	782 (37.5/86.7)	2083 (100/86.1)
Total	468 (19.4/100)	1049 (43.4/100)	902 (37.3/100)	2419 (100/100)

**COVID-19 vaccination status of the surroundings**				
Around 0–25 %	98 (23.3/20.9)	182 (43.3/17.4)	140 (33.3/15.5)	420 (100/17.4)
Around 50 %	127 (25.6/27.1)	185 (37.2/17.6)	185 (37.2/20.5)	497 (100/21.0)
Around 75 %	180 (19.0/39.0)	399 (42.1/38.0)	368 (38.9/40.8)	947 (100/39.2)
Around 100 %	63 (11.4/13.5)	283 (51.0/27.0)	209 (37.7/23.2)	555 (100/23.0)
Total	468 (19.4/100)	1049 (43.4/100)	902 (37.3/100)	2419 (100/100)

***Psychological characteristics***				
**Benefits of COVID-19 vaccination**				
Insignificant	145 (54.7/31.0)	79 (29.8/7.53)	41 (15.5/4.6)	265 (100/11.0)
Significant	114 (7.9/24.4)	790 (54.8/75.3)	539 (37.4/59.8)	1443 (100/60.0)
Neutral	209 (29.4/44.7)	180 (25.3/17.2)	322 (45.3/35.7)	711 (100/29.4)
Total	468 (19.4/100)	1049 (43.4/100)	902 (37.3/100)	2419 (100/100)

**Risks of COVID-19 vaccination**				
Insignificant	71 (8.4/15.2)	538 (64.0/51.3)	232 (27.6/25.7)	841 (100/34.8)
Significant	189 (36.3/40.4)	182 (34.9/17.4)	150 (28.8/16.6)	521 (100/21.5)
Neutral	208 (19.7/44.4)	329 (31.1/31.4)	520 (49.2/57.7)	1057 (100/43.7)
Total	468 (19.4/100)	1049 (43.4/100)	902 (37.3/100)	2419 (100/100)

**Trust in public authorities**				
Disagree	191 (51.2/40.8)	93 (24.9/8.9)	89 (23.9/9.9)	373 (100/15.4)
Agree	85 (7.5/18.2)	669 (59.1/63.8)	378 (33.4/41.9)	1132 (100/46.8)
Neutral	192 (21.0/41.0)	287 (31.4/27.4)	435 (47.6/48.2)	914 (100/37.8)
Total	468 (19.4/100)	1049 (43.4/100)	902 (37.3/100)	2419 (100/100)

**Perceived peer Influence**				
Disagree	180 (55.2/38.5)	71 (21.8/6.8)	75 (23.0/8.3)	326 (100/13.5)
Agree	125 (9.05/26.7)	788 (57.1/75.1)	468 (33.9/51.9)	1381 (100/57.1)
Neutral	163 (22.9/34.8)	190 (26.7/18.1)	359 (50.4/39.8)	712 (100/29.4)
Total	468 (19.4/100)	1049 (43.4/100)	902 (37.3/100)	2419 (100/100)

**COVID-19 anxiety**				
I'm not worried at all	93 (34.2/19.9)	101 (37.1/9.6)	78 (28.7/8.7)	272 (100/11.2)
Somewhat concerned	244 (17.6/52.1)	600 (43.3/57.2)	543 (39.2/60.2)	1387 (100/57.3)
Anxious	101 (17.5/21.6)	255 (44.2/24.3)	221 (38.3/24.5)	577 (100/23.9)
Fearful and anxious	30 (16.4/6.4)	93 (50.8/8.9)	60 (32.8/6.7)	183 (100/7.57)
Total	468 (19.4/100)	1049 (43.4/100)	902 (37.3/100)	2419 (100/100)

**Disruption to daily life**				
Not at all or not much	151 (19.6/32.3)	353 (45.7/33.7)	268 (34.7/29.7)	772 (100/31.9)
To some extent	213 (17.8/45.5)	510 (42.5/48.6)	477 (39.8/52.9)	1200 (100/49.6)
Yes	104 (23.3/22.2)	186 (41.6/17.7)	157 (35.1/17.4)	447 (100/18.5)
Total	468 (19.4/100)	1049 (43.4/100)	902 (37.3/100)	2419 (100/100)

***Information sources regarding COVID-19***				
**Trust specialists**				
Mean [20]	2.2 (0.6)	2.5 (0.7)	2.4 (0.6)	2.4 (0.6)

**Trust mass media**				
Mean [20]	2.0 (0.7)	2.4 (0.7)	2.3 (0.6)	2.3 (0.7)

**Trust SNS websites**				
Mean [20]	1.8 (0.6)	1.9 (0.6)	1.8 (0.6)	1.8 (0.6)

**Trust government**				
Mean [20]	2.0 (0.7)	2.5 (0.7)	2.3 (0.7)	2.3 (0.7)

**Trust close people**				
Mean [20]	2.1 (0.7)	2.3 (0.7)	2.2 (0.6)	2.2 (0.7)

**The number of information sources utilized for COVID-19**				
Mean [20]	9.8 (9.2)	14.5 (9.6)	13.6 (9.9)	13.2 (9.8)

SD: standard deviation

### 3.3 Health-related characteristics

About half (50.6 %) of parents had received some or all of their routine vaccinations (50.6 %). 86.1 % of respondents indicated that they themselves had received or planned to receive COVID-19 vaccination. In terms of the COVID-19 vaccination status of their surrounding population, the most frequent response was around 75 % (39.2 %), while the second most frequent response was around 100 % (23.0 %).

### 3.4 Psychological characteristics

Of the participants, 59.7 % recognized the significant benefits of vaccination, while 21.5 % recognized significant risks. In response to the question of whether public institutions can be trusted regarding vaccine administration, 46.8 % responded “Agree”. The proportion of participants who believed that they should be vaccinated based on the vaccination status of others was 57.1 %. Furthermore, 88.8 % of the respondents reported some kind of anxiety about COVID-19, 68.1 % of the respondents reported that COVID-19 disrupted their daily life to some extent or more.

### 3.5 Information sources regarding COVID-19

The mean score on a scale of 1–4 for trust in healthcare professionals was 2.4; for mass media 2.3; for SNS and websites 1.8; for government agencies 2.3; for close people 2.2. The mean total number of referenced information resources was 13.2 out of 30 options. The frequency data on the sources of information for this study was not collected.

### 3.6 Factors associated with the willingness of parents who responded “agree” to COVID-19 vaccination for children

The results of the regression model are presented in Table 2 and Supplementary Table 4. After adjustment by multinomial logistic regression, several variables were found to be statistically significant among those who answered “agree” regarding parental willingness for children's COVID-19 vaccination. Firstly, sociodemographic variables including gender (Men in reference to women: OR 1.55; 95 % CI 1.12–2.17), highest educational level (Junior College/Vocational in reference to under high school: OR 0.61; 95 % CI 0.40–0.94, Bachelor’s /Master’s/Doctoral degree in reference to under high school: OR 0.60; 95 % CI 0.42–0.86) and occupation (Student/Homemaker in reference to Medical and Healthcare: OR 0.50; 95 % CI 0.29–0.86) were statistically significant; meaning men are more likely to be willing to vaccinate their children against COVID-19 compared to women, while parents with higher education and student and homemaker are less likely to be willing to do so compared to lower education and healthcare workers respectively.

**Table 2:** **Factors Associated with Parental Hesitation on COVID-19 Vaccination for Children.** (*Adjusted for other variables (Please refer to Supplementary Table 4 for all variables)).

	**Agree**		**Not sure**	
	OR (95 % conf.interval)	*p*-value	OR (95 % conf.interval)	*p*-value
***Sociodemographic characteristics***				
**Gender**				
Women	1 (Ref. group)		1 (Ref. group)	
Men	1.55 (1.12–2.17)	0.01	1.09 (0.79–1.50)	0.60

**Highest educational level**				
Middle/High school	1 (Ref. group)		1 (Ref. group)	
Junior College/vocational	0.61 (0.40––0.94)	0.02	0.62 (0.42–0.93)	0.02
Bachelor's/Master's/Doctoral	0.60 (0.42–0.86)	0.01	0.59 (0.42–0.82)	<0.01

**Occupation**				
Medical and Healthcare	1 (Ref. group)		1 (Ref. group)	
Manufacturing	1.09 (0.61––1.96)	0.77	0.88 (0.50–1.56)	0.66
Student/Homemaker	0.50 (0.29–0.86)	0.01	0.73 (0.44–1.21)	0.22
Other	0.94 (0.57--1.55)	0.82	0.87 (0.54–1.41)	0.57

***Health related factors***				
**Routine vaccinations**				
Yes	0.75 (0.53–1.06)	0.11	0.69 (0.49–0.98)	0.04
Some of them	1.08 (0.72–1.62)	0.72	0.78 (0.52–1.17)	0.22
No	1 (Ref. group)		1 (Ref. group)	
Not sure	0.89 (0.59–1.36)	0.60	1.26 (0.85–1.86)	0.25

**COVID-19 vaccination status**				
No	1 (Ref. group)		1 (Ref. group)	
Yes or Will	3.48 (2.25–5.38)	<0.01	1.29 (0.91–1.84)	0.16

**COVID-19 vaccination status of the surroundings**				
Around 0–25 %	1 (Ref. group)		1 (Ref. group)	
Around 50 %	1.05 (0.69–1.59)	0.82	1.17 (0.78–1.74)	0.45
Around 75 %	1.13 (0.78–1.65)	0.52	1.30 (0.90–1.88)	0.16
Around 100 %	1.84 (1.18–2.88)	0.01	2.00 (1.29–3.10)	<0.01

***Psychological factors***				
**Benefits of COVID-19 vaccination**				
Insignificant	1 (Ref. group)		1 (Ref. group)	
Significant	2.05 (1.27–3.30)	<0.01	5.08 (3.09–8.30)	<0.01
Neutral	0.73 (0.45–1.17)	0.19	1.79 (1.11–2.89)	0.02

**Risks of COVID-19 vaccination**				
Insignificant	1 (Ref. group)		1 (Ref. group)	
Significant	0.29 (0.19–0.43)	<0.01	0.48 (0.32–0.71)	<0.01
Neutral	0.48 (0.33–0.71)	<0.01	1.14 (0.77–1.68)	0.52

**Trust in public authorities**				
Disagree	1 (Ref. group)		1 (Ref. group)	
Agree	3.23 (2.04–5.11)	<0.01	2.78 (1.79–4.32)	<0.01
Neutral	1.65 (1.05–2.59)	0.03	2.06 (1.43–2.98)	<0.01

**Perceived peer influence**				
Disagree	1 (Ref. group)		1 (Ref. group)	
Agree	3.23 (2.04–5.11)	<0.01	1.96 (1.26–3.03)	<0.01
Neutral	1.65 (1.05–2.59)	0.03	2.13 (1.41–3.19)	<0.01

**COVID-19 anxiety**				
I'm not worried at all	1 (Ref. group)		1 (Ref. group)	
Somewhat concerned	1.36 (0.88–2.10)	0.16	1.63 (1.07–2.48)	0.02
Anxious	1.30 (0.79–2.15)	0.31	1.55 (0.95–2.52)	0.08
Fearful and anxious	3.17 (1.61–6.25)	0.00	2.25 (1.15–4.37)	0.02

**Disruption to daily life**				
Not at all or not much	1 (Ref. group)		1 (Ref. group)	
To some extent	0.66 (0.47–0.91)	0.01	0.82 (0.60–1.13)	0.23
Yes	0.45 (0.29–0.68)	<0.01	0.59 (0.39–0.88)	0.01

***Information related factors***				
**Trust in information sources**				
Trust specialists	1.27 (0.95–1.70)	0.11	1.06 (0.80–1.41)	0.70
Trust mass media	1.25 (0.94–1.68)	0.13	1.24 (0.92–1.65)	0.15
Trust SNS websites	0.86 (0.62–1.18)	0.35	0.60 (0.44–0.83)	<0.01
Trust government	1.04 (0.78–1.39)	0.79	1.03 (0.78–1.38)	0.82
Trust close people	0.86 (0.67–1.11)	0.26	0.94 (0.74–1.21)	0.65

**The number of information sources utilized for COVID-19**				
	1.02 (1.01–1.04)	0.01	1.03 (1.01–1.04)	<0.01

Among the health-related variables, COVID-19 vaccination status (Yes or Will in reference to No: OR 3.48; 95 % CI 2.25–5.38) and COVID-19 vaccination status of the surroundings (Around 100 % in reference to Around 0–25 %: OR 1.84; 95 % CI 1.18–2.88) were statistically significant; meaning parents who have either received the COVID-19 vaccine at least once or express a willingness to do so and parents who reside in an area with a high COVID-19 vaccination rate are more likely to exhibit a high inclination to vaccinate their children.

Regarding psychological factors, perception of benefits of COVID-19 vaccination (Significant in reference to Insignificant: OR 2.05; 95 % CI 1.27–3.30), perception of risks of COVID-19 vaccination (Significant in reference to Insignificant: OR 0.29; 95 % CI 0.19–0.43, Neutral in reference to Insignificant: OR 0.48; 95 % CI 0.33–0.71), trust in public authorities (Agree in reference to Disagree: OR 3.23; 95 % CI 2.04–5.11, Neutral in reference to Disagree: OR 1.65; 95 % CI 1.05–2.59), perceived peer influence (Agree in reference to Disagree: OR 3.23; 95 % CI 2.04–5.11, Neutral in reference to Disagree: OR 1.65; 95 % CI 0.1.05–2.59), COVID-19 anxiety (Fearful and anxious in reference to I'm not worried at all: OR 3.17; 95 % CI 1.61–6.25) and disruption to daily life (To some extent in reference to Not at all or not much: OR 0.66; 95 % CI 0.47–0.91, Yes in reference to Not at all or not much: OR 0.45; 95 % CI 0.29–0.68) were found to be associated; meaning individuals who significantly perceive the benefits of the COVID-19 vaccine, have a trust in government administration, believe that they should get vaccinated if those around them have already received COVID-19 vaccine, and have strong anxiety for COVID-19 tend to exhibit a high willingness to vaccinate their children. On the other hand, individuals who are aware of the risks of the COVID-19 vaccine and those who reported experiencing disruptions in their lives due to COVID-19 tend to have a lower willingness to vaccinate their children.

In addition, the number of referenced information sources on COVID-19 was related to a supportive attitude towards children’s vaccination (OR 1.02 for each additional referenced information source; 95 % CI 1.01–1.04).

### 3.7 Factors associated with the willingness of parents who responded “not sure” to COVID-19 vaccination for children

For those who responded “not sure”, the following variables were found to be associated. Firstly, regarding sociodemographic variables, highest educational level (Junior College /Vocational in reference to under high school: OR 0.62; 95 % CI 0.42–0.93, Bachelor’s /Master’s/Doctoral degree in reference to under high school: OR 0.59; 95 % CI 0.42–0.82) was statistically significant, meaning parents with higher education have lower willingness to get children vaccinated.

In terms of health related factors, routine vaccinations (Yes in reference to No: OR 0.69; 95 % CI 0.49–0.98) and COVID-19 vaccination status of the surroundings (Around 100 % in reference to Around 0–25 %: OR2.00; 95 % CI 1.29–3.10) were statistically significant; meaning that parents who receive their own routine vaccinations tend to have a lower willingness to vaccinate children, and that parents who perceived that their communities had a higher vaccination rate are more likely to be unsure about vaccinating their children rather than disagreeing it.

Regarding psychological factors, individuals who perceive the benefits of the COVID-19 vaccine as significant (Significant in reference to Insignificant: OR 5.08; 95 % CI 3.09–8.30, Neutral in reference to Insignificant: OR 1.79; 95 % CI 1.11–2.89), have a trust in government administration (Agree in reference to Disagree: OR 2.78; 95 % CI 0.1.79–4.32, Neutral in reference to Disagree: OR 2.06; 95 % CI 1.43–2.98), believe that they should get vaccinated if those around them have already received the COVID-19 vaccine (Agree in reference to Disagree: OR 1.96; 95 % CI 1.26–3.03, Neutral in reference to Disagree: OR2.13; 95 % CI 1.41–3.19), and have anxiety for COVID-19 (Somewhat concerned in reference to I'm not worried at all: OR1.63; 95 % CI 1.07–2.48, Fearful and anxious in reference to I'm not worried at all: OR 2.25; 95 % CI 1.15–4.37) tend to exhibit uncertainty about vaccinating children. On the other hand, individuals who are aware of the risks of the COVID-19 vaccine strongly (Significant in reference to Insignificant: OR 0.48; 95 % CI 0.32–0.71) and those who reported experiencing disruptions in their lives due to COVID-19 (Yes in reference to Not at all or not much: OR 0.59; 95 % CI 0.39–0.88) tend to have a higher likelihood to disagree with the childhood vaccination.

In addition, people who trust social media and websites as COVID-19 information source showed higher disagreement to get children vaccinated against COVID-19 (OR 0.60; 95 % CI 0.44–0.83), while the number of referenced information sources utilized for COVID-19 was related to more unsure attitude towards children’s vaccination (OR 1.03 for each additional referenced information source; 95 % CI 1.01–1.04).

## 4 Discussion

### 4.1 Outcome distribution

As contextual background, on the commencement date of the survey, Japan achieved 75.8 % vaccination coverage among the total population for the initial COVID-19 vaccine dose [22], [23], meaning that the public was relatively accepting the vaccines in general. However, less than half of the parents answered “agree” to get their children vaccinated against COVID-19 in this study. Parental vaccination willingness for their children is involved with various context-specific factors and it can vary depending on vaccine type, time, and social contexts where a new vaccine was introduced [24]. Reflecting on its history, while DPT vaccines were well accepted among parents [25], concerns about adverse events sometimes led to the public negative sentiments around vaccines and the increased parental vaccine hesitancy, starting with pertussis vaccination in the 1970s and later with rubella vaccination in the late 1980s, and more recently with the HPV vaccine [25].

### 4.2 Sociodemographic predictors of parental willingness towards COVID-19 vaccination for children

The results demonstrate that women tend to exhibit a more cautious attitude toward vaccinating children, which aligns with prior research conducted both within Japan and internationally [8], [26], [27]. There is research which has suggested that women exhibit higher antibody responses and report a greater number of adverse reactions following preventive vaccinations compared to males [28]. Having negative past experiences might influence cautious decision-making among women. Research investigating gender differences in decision-making concerning risk has also revealed that females tend to invest more time in evaluating risks compared to males [29]. Furthermore, a previous international study on parental willingness to vaccinate children against COVID-19 indicated that differences between mothers and fathers in decision-making might stem from mothers' proactive role in children's healthcare and active involvement in healthcare decisions [30]. This background may have also contributed to the more cautious attitude among women.

In terms of educational level, individuals with educational attainment beyond high school, specifically those with bachelor's degree or higher, displayed a statistically significant lower level of willingness regarding children's vaccination. Prior research conducted both in Japan and internationally indicated conflicting results regarding the association between educational levels and vaccination willingness. Some studies showed that higher educational attainment were associated with a greater intention to vaccinate children against COVID-19 [6], [31], [32], [33], [34] and others indicated that, especially in high-income countries, a higher level of education was associated with a tendency to refuse self-vaccination [35], [36]. These inconsistent findings imply that the role of educational level in shaping parental vaccination decision-making varies depending on contexts in which they decide about the vaccination. The survey was conducted just before the commencement of children's COVID-19 vaccination, and at that time, the public was exposed to both correct information and misinformation about these new types of vaccines for children through offline and online platforms. This disparity in available information may have influenced the observed variations in results [37]. In previous research, there is an indication that individuals with higher educational qualifications tend to exhibit a higher level of literacy in terms of information gathering related to vaccines [38]. When both positive and negative information on new vaccines is circulated, individuals who have higher educational attainment tend to base their decisions on a careful accumulation of information and, thus, they may display a heightened sense of caution when it comes to vaccinating their children.

### 4.3 Health-related psychological predictors of parental willingness towards COVID-19

When comparing “disagree” and “not sure” group, individuals who reported receiving all their routine vaccinations tended to answer “disagree” than “not sure” regarding children’s COVID-19 vaccination compared to those who did not receive all their routine vaccinations. Moreover, although not statistically significant, the results within the group that responded “agree” also indicated similar result. These results contrast with prior research in the United States which found a positive association between having received other vaccines and an inclination to vaccinate their children against COVID-19 [39].The divergent outcomes in this study may be attributed to the fact that at the time of the study, COVID-19 vaccination had only recently become a feasible and practical choice. Similar to the aforementioned discourse on educational background, this timing could have influenced individuals who were in a state of uncertainty (not sure or no) to approach the decision-making process more cautiously. Particularly, individuals with strong health consciousness, who regularly received routine vaccinations, might be more vigilant to a new technology in healthcare, such as RNA vaccines, and might not want to take any risks of potentially damaging their health by being vaccinated with the new type of vaccine.

Regarding the vaccination status of those around parents, in both “agree” and “not sure” groups, those who reported 100 % vaccination status were more motivated to have children vaccinated. Prior research investigating vaccination acceptance through the lens of Social Contagion Theory suggests that social networks wield a significant influence in shaping both favorable and unfavorable attitudes, as well as vaccination coverage. These networks may likewise exert a comparable impact on parental intentions concerning their children's COVID-19 immunization [40].

### 4.4 Psychological predictors of parental willingness towards COVID-19 vaccination for children

People who perceive the benefits of COVID-19 vaccination tend to have a higher willingness to vaccinate children, whereas those who recognize the risks exhibit a lower inclination for child vaccination. Particularly within the group that responded with “agree” individuals who perceive risks as “significant” show a notably reduced willingness for vaccination. Even among those who responded with “neutral,” in contrast to the benefits, there is a statistically significant decrease in willingness for vaccination regarding the drawbacks. In prior research conducted in Japan, investigating parental COVID-19 vaccination intention for children revealed that factors influencing parental low willingness to be vaccinated leaned more heavily toward concerns about adverse reactions rather than vaccine effectiveness [6]. Other previous research also indicates that fear of vaccine side effects is a major reason for hesitation in vaccinating children [41].

Higher trust in public institutions was associated with greater willingness to be vaccinated. In prior research investigating the relationship between one's own COVID-19 vaccination intention and trust in public institutions, it was found that trust in the government's response to the pandemic positively influenced vaccination willingness [31]. This finding can be extended to parental vaccination intentions for children.

Regarding COVID-19 anxiety, individuals who responded as “fearful and anxious” exhibited a higher willingness for vaccination in both the “agree” groups. In previous Japanese studies, it has been indicated that groups with low COVID-19 anxiety tend to take fewer preventive actions and have limited access to information [42]. Conversely individuals who express strong anxiety may be inclined to actively seek information and engage in preventive behaviors, including vaccination for children.

### 4.5 Information-related predictors of parental willingness towards COVID-19 vaccination for children

Firstly, “agree” group exhibited high trust in information sources from specialists, such as healthcare providers. Previous studies also indicates that parents who did not receive information from healthcare providers were more hesitant towards vaccines [43]. Additionally, other previous studies have shown that parents who use healthcare professionals as their information source are more likely to have a higher willingness to vaccinate their children, highlighting the importance of information provided by medical professionals [44], [45].

When comparing “disagree” group and “not sure” group, the results indicated that individuals who used social media as a trusted information source tend to answer “disagree”. In a study conducted in Japan investigating parental COVID-19 vaccination intention for children, similar findings where those who relied on social media as their most trusted source of information exhibited lower vaccination intent compared to those who utilized official sources of information [8]. Moreover, prior research has identified social media as a primary source of information for parents and a major cause of hesitation [20]. Additionally, other researches indicate that increased misinformation on social media correlates with a decline in users' vaccination intent, showing a consistent pattern across different regions [31], [46]. These studies suggest that misinformation is more likely to circulate on social media platforms, which contributes to the observed link between these information sources and vaccine hesitancy.

Conversely, the study also found a significant positive relationship between a higher number of information sources and vaccination intent. This suggests that utilizing a diverse range of information sources beyond personal-driven platforms like social media may enhance individuals' resilience against misinformation and subsequently increase vaccination intent. Furthermore, prior Japanese research indicates that individuals with higher health literacy tend to gather information from multiple sources when it comes to COVID-19 [47]. This observation highlights the connection between robustness against misinformation and the utilization of multiple information sources. Furthermore, prior research has indicated that the number of information sources used is positively correlated with the level of acceptance of preventive behaviors against COVID-19 [48].

### 4.6 Implications

This study revealed that various factors, including socioeconomic background, health considerations, and information sources, collectively impact vaccination intent.

These findings suggest three effective policy approaches. Firstly, the government should promote comprehensive health literacy education for parents to encourage informed decision-making and caution against misinformation on social media. This initiative aims to equip parents with skills for accurate health information gathering, assessment, and judgment. Prior research indicates that comprehensive health education is inadequately integrated into Japan's formal curriculum, limiting opportunities to enhance these skills. [49], [50].

Second, government authorities should prioritize providing comprehensive information on vaccination, including potential risks, from a wide array of sources, including social media platforms, to enhance resilience against misinformation among parents.

Thirdly, governmental agencies should promote effective risk communication strategies to enhance parental trust. Strengthening the vaccine risk assessment system and expanding ways to convey potential drawbacks, including adverse reactions, is crucial. Providing timely and accurate information can enhance trust in government agencies and improve vaccination willingness.

## 4.7 Limitations

Online surveys have potential biases, including a participant pool limited to those with internet access and an overrepresentation of individuals highly interested in COVID-19 vaccines due to the first-come, first-served quota sampling. Additionally, parents' reported vaccination intentions may not reflect their actual actions. The sample's representativeness showed limitations due to significant sociodemographic differences between respondents and non-respondents in the second survey, indicating potential response bias (see Supplementary Table 5). Conducted before COVID-19 vaccines for children, the study's findings may not be fully generalizable but are valuable for future pandemics. Recall bias is possible since the survey relied on respondents' recollections, though most questions focused on sociodemographic items and vaccination opinions, minimizing memory errors. Additionally, potential social desirability bias was likely limited due to the lack of fixed societal opinions and scarce information about COVID-19 vaccinations at the time. Lastly, the cross-sectional study design makes drawing causal conclusions challenging.

## 5 Conclusions

This study examined factors influencing parental intentions for pediatric COVID-19 vaccination in Japan during the introduction of the vaccine. Key factors included socioeconomic background, health status, and psychological factors, with recognition of vaccine risks being crucial. Higher education and routine vaccination did not increase vaccination intent, likely due to caution about side effects. Effective risk communication by government bodies can address public concerns about new vaccines. While social media may hinder vaccination willingness, access to multiple reliable information sources strengthens intent. Efforts to provide diverse information channels and enhance health literacy among parents are essential for promoting accurate vaccine decision-making.

## Author contributions

Conception/design of the work: MU, SN; drafting of the work: MU; acquisition of data: KMS, HM, SN; analysis of data: MU, AC, SN; interpretation of findings: all authors; substantially revised the work: all authors.

## Institutional review board statement

Ethical approval was granted by the Ethics Committee of Keio University School of Medicine under authorization number 20200340.

## Informed consent statement

Respondents had to provide their consent before they proceeded to the questionnaire response page.

## Funding

The present work was supported in part by a grant from the Kanagawa Prefectural Government of Japan, AIST government subsidies, and the Precursory Research for Embryonic Science and Technology from the Japan Science and Technology Agency (JPMJPR22R8).

## CRediT authorship contribution statement

**Mami Ueta:** Writing – original draft, Visualization, Project administration, Investigation, Formal analysis, Data curation, Conceptualization. **Alton Cao:** Writing – review & editing. **Michio Murakami:** Writing – review & editing. **Hana Tomoi:** Writing – review & editing. **Stuart Gilmour:** Writing – review & editing. **Keiko Maruyama-Sakurai:** Writing – review & editing. **Yoshihiro Takayama:** Writing – review & editing. **Yoshitake Takebayashi:** Writing – review & editing. **Masahiro Hashizume:** Writing – review & editing. **Rauniyar Santosh Kumar:** Writing – review & editing. **Hiroyuki Kunishima:** Writing – review & editing. **Wataru Naito:** Writing – review & editing. **Tetsuo Yasutaka:** Writing – review & editing. **Satoshi Kaneko:** Writing – review & editing. **Hiroaki Miyata:** Data curation. **Shuhei Nomura:** Writing – review & editing, Supervision, Data curation, Conceptualization.

## Declaration of competing interest

The authors declare that they have no known competing financial interests or personal relationships that could have appeared to influence the work reported in this paper.

## Data Availability

The datasets generated and/or analyzed during the current study are not publicly available due to ethical considerations but are available from the corresponding author on reasonable request.

## References

[1] Ministry of Health, Labour and Welfare, *Why is the “mandatory effort” now applied to the vaccination of children (5-11 years old)? (Japanese)*. 2022, Ministry of Health, Labour and Welfare; Tokyo.

[2] Ministry of Health, Labour and Welfare, *Does the “duty of effort” apply to Covid-19 vaccination of infants (6 months to 4 years of age)? (Japanese)*. 2022, Ministry of Health, Labour and Welfare; Tokyo.

[3] Japan Pediatric Society, *Views on vaccination of children between 6 months and 5 years of age with the vaccine for Covid-19 (Japanese)*. 2022, Committee on Immunization and Infectious Disease Control, Japan Pediatric Society; Tokyo.

[4] Japan Pediatric Society, *Views on vaccination of children aged 5-17 years with the vaccine for Covid-19 (Japanese)*. 2022, Japan Pediatric Society; Tokyo.

[5] Prime Ministers' Office in Japan, *Immunization results by age group (Japanese)*. 2023, Prime ministers' office in Japan; Tokyo.

[6] Yoda T. (2021). Parents’ hesitation about getting their children vaccinated against COVID-19 in Japan. Hum Vaccin Immunother.

[7] Plotkin S.A. (2021). Considering Mandatory Vaccination of Children for COVID-19. Pediatrics.

[8] Horiuchi S. (2021). Factors of parental COVID-19 vaccine hesitancy: A cross sectional study in Japan. PLoS One.

[9] Simmons L.A. (2022). Understanding COVID-19 vaccine uptake during pregnancy: 'Hesitance', knowledge, and evidence-based decision-making. Vaccine.

[10] Cross Marketing Inc, *Cross Marketing*. 2023, Cross Marketing Inc,,; Tokyo.

[11] Lin C. (2020). Confidence and Receptivity for COVID-19 Vaccines: A Rapid Systematic Review. Vaccines (Basel).

[12] Robinson E. (2021). International estimates of intended uptake and refusal of COVID-19 vaccines: A rapid systematic review and meta-analysis of large nationally representative samples. Vaccine.

[13] Statistics Bureau, Ministry of Internal Affairs and Communications, *Population Census 2015 Statistical Maps of Japan (Japanese)*. 2018, Statistics Bureau, Ministry of Internal Affairs and Communications; Tokyo.

[14] Adachi M. (2022). Factors associated with the risk perception of COVID-19 infection and severe illness: A cross-sectional study in Japan. SSM Popul Health.

[15] Nomura S. (2021). Reasons for being unsure or unwilling regarding intention to take vaccine among Japanese people: A large cross-sectional national survey. Lancet Reg Health West Pac.

[16] Yoneoka D. (2022). Identification of optimum combinations of media channels for approaching COVID-19 vaccine unsure and unwilling groups in Japan. Lancet Reg Health West Pac.

[17] Nomura, S., et al., Characterizing reasons for reversals of COVID-19 vaccination hesitancy among Japanese people: One-year follow-up survey Lancet Reg Health West Pac, 2022. **27**: p.100541. 10.1016/j.lanwpc.2022.100541 PMC9302916 35892010

[18] Task Force on Covid-19, *Public Notice on the Revision of the Public Notice on Priority Measures for the Prevention of the Spread of COVID-19*. 2022, Task Force on COVID-19.

[19] Task Force on Covid-19, *Basic Coping Policies for New Covid-*2022, Task Force on COVID-19,; Tokyo.

[20] Almuqbil M. (2023). Parental COVID-19 Vaccine Hesitancy for Children and Its Influencing Factors: A Riyadh-Based Cross-Sectional Study. Vaccines (Basel).

[21] Mccuen R.H. (2003).

[22] Ministry of Health, Labour and Welfare, *Current status of new coronavirus infection and response by the Ministry of Health, Labour and Welfare (February 4, 2022 version) (Japanese)*. 2022, Ministry of Health, Labour and Welfare; Tokyo.

[23] Digital Agency, *COVID-19 vaccination status*. 2022, Digital Agency; Tokyo.

[24] World Health Organization, t. S. W. G. o. V. H., *REPORT OF THE SAGE WORKING GROUP ON VACCINE HESITANCY.* Geneva2014.

[25] Andrew Gordon M.R.R. (2021). The Puzzle of Vaccine Hesitancy in Japan. J Japanese Stud Soc Jpn Stud.

[26] Yigit M. (2021). Evaluation of COVID-19 Vaccine Refusal in Parents. Pediatr Infect Dis J.

[27] Skjefte M. (2021). COVID-19 vaccine acceptance among pregnant women and mothers of young children: results of a survey in 16 countries. Eur J Epidemiol.

[28] Gebhard C. (2020). Impact of sex and gender on COVID-19 outcomes in Europe. Biol Sex Differ.

[29] Cordina M. (2021). Attitudes towards COVID-19 vaccination, vaccine hesitancy and intention to take the vaccine. Pharm Pract (Granada).

[30] Goldman R.D. (2022). Parental gender differences in attitudes and willingness to vaccinate against COVID-19. J Paediatr Child Health.

[31] Jennings W. (2021). Lack of Trust, Conspiracy Beliefs, and Social Media Use Predict COVID-19 Vaccine Hesitancy. Vaccines (Basel).

[32] Okamoto S. (2022). COVID-19 vaccine hesitancy and vaccine passports: a cross-sectional conjoint experiment in Japan. BMJ Open.

[33] Temsah M.H. (2021). Parental Attitudes and Hesitancy About COVID-19 vs. Routine Childhood Vaccinations: A National Survey. Front Public Health.

[34] Wang B. (2021). COVID-19 Immunisation, Willingness to Be Vaccinated and Vaccination Strategies to Improve Vaccine Uptake in Australia. Vaccines (Basel).

[35] Bergen N. (2023). Global state of education-related inequality in COVID-19 vaccine coverage, structural barriers, vaccine hesitancy, and vaccine refusal: findings from the Global COVID-19 Trends and Impact Survey. Lancet Glob Health.

[36] Loomba S. (2021). Measuring the impact of COVID-19 vaccine misinformation on vaccination intent in the UK and USA. Nat Hum Behav.

[37] Okui T. (2021). An analysis of health inequalities depending on educational level using nationally representative survey data in Japan, 2019. BMC Public Health.

[38] Fenta E.T. (2023). Health literacy and COVID-19 vaccine acceptance worldwide: A systematic review. SAGE Open Med.

[39] Pingali C, Z. F., Santibanez TA, Elam-Evans LD, Hill HA, Valier MR, Singleton JA. *Associations Between Routine Adolescent Vaccination Status and Parental Intent to Get a COVID-19 Vaccine for Their Adolescent*. 10.1001/jamapediatrics.2022.4877 PMC9857233 36508203

[40] Konstantinou P. (2021). Transmission of Vaccination Attitudes and Uptake Based on Social Contagion Theory: A Scoping Review. Vaccines (Basel).

[41] Iqbal M.S. (2023). Vaccine Hesitancy of COVID-19 among Parents for Their Children in Middle Eastern Countries-A Systematic Review. Vaccines (Basel).

[42] Shiina A. (2020). Relationship between perception and anxiety about COVID-19 infection and risk behaviors for spreading infection: A national survey in Japan. Brain Behav Immun Health.

[43] Napoli A. (2022). Parents' reasons to vaccinate their children aged 5–11 years against COVID-19 in Italy. Front Med (Lausanne).

[44] Miraglia Del Giudice G. (2023). Willingness and hesitancy of parents to vaccinate against COVID-19 their children ages 6 months to 4 years with frail conditions in Italy. Front Public Health.

[45] Wang Y. (2023). Key factors influencing paediatric COVID-19 vaccine hesitancy: a brief overview and Decision-making Trial and Evaluation Laboratory analysis. Public Health.

[46] Wilson S.L. (2020). *Social media and vaccine hesitancy.* BMJ Glob. Health.

[47] Inoue M. (2022). The Relationship Between Information Sources, Health Literacy, and COVID-19 Knowledge in the COVID-19 Infodemic: Cross-sectional Online Study in Japan. J Med Internet Res.

[48] Uchibori M. (2022). Preventive Behaviors and Information Sources during COVID-19 Pandemic: A Cross-Sectional Study in Japan. Int J Environ Res Public Health.

[49] Nakayama K. (2022). Associations between health literacy and information-evaluation and decision-making skills in Japanese adults. BMC Public Health.

[50] Nakayama K. (2015). Comprehensive health literacy in Japan is lower than in Europe: a validated Japanese-language assessment of health literacy. BMC Public Health.

