# Supplementary material for: Factors influencing parental COVID-19 vaccination willingness for children in Japan
Source: Vaccine X. 2024 Jul 18;19:100528. doi: 10.1016/j.jvacx.2024.100528 (PMC11332198; doi:10.1016/j.jvacx.2024.100528)
Supplement: Supplementary Data 1 [file mmc1.docx]

*Supplementary Table 1: Survey Questions in Japanese with English Translations*

| Questions | Answer choices |
| --- | --- |
| Outcome |  |
| 新型コロナウイルス感染症のワクチンについて、今後、１２歳未満の子どもも接種できるようになった場合、あなたは次のどれにあてはまりますか？（ＳＡ）  When it becomes possible for children under 12 to receive the COVID-19 vaccine in the future, which of the following applies to you? (SA) | 賛成（該当する年齢の子どもがいる）  Agree (have children of the relevant age)  賛成（該当する年齢の子どもはいない）Agree (have no children of the relevant age)  反対（該当する年齢の子どもがいる）Disagree (have children of the relevant age)  反対（該当する年齢の子どもはいない）Disagree (have no children of the relevant age)  どちらとも言えない  Not sure (have children of the relevant age)  どちらとも言えない  Not sure (have no children of the relevant age) |
| Sociodemographic Questions |  |
| あなたのお住まいの都道府県を教えてください。（ＳＡ）  Which prefecture do you live in? (SA) | 北海道 Hokkaido 青森県 Aomori 岩手県 Iwate 宮城県 Miyagi 秋田県 Akita 山形県 Yamagata 福島県 Fukushima 茨城県 Ibaraki 栃木県 Tochigi 群馬県 Gunma 埼玉県 Saitama 千葉県 Chiba 東京都 Tokyo 神奈川県 Kanagawa 新潟県 Niigata 富山県 Toyama 石川県 Ishikawa 福井県 Fukui 山梨県 Yamanashi 長野県 Nagano 岐阜県 Gifu 静岡県 Shizuoka 愛知県 Aichi 三重県 Mie 滋賀県 Shiga 京都府 Kyoto 大阪府 Osaka 兵庫県 Hyogo 奈良県 Nara 和歌山県 Wakayama 鳥取県 Tottori 島根県 Shimane 岡山県 Okayama 広島県 Hiroshima 山口県 Yamaguchi 徳島県 Tokushima 香川県 Kagawa 愛媛県 Ehime 高知県 Kochi 福岡県 Fukuoka 佐賀県 Saga 長崎県 Nagasaki 熊本県 Kumamoto 大分県 Oita 宮崎県 Miyazaki 鹿児島県 Kagoshima 沖縄県 Okinawa |
| あなたの性別を教えてください。※自分としての認識でお答えください。（ＳＡ）  What is your gender? Please answer by your personal perception. (SA) | 女性 Women 男性 Men その他 Other |
| あなたの年齢を教えてください。／歳（ＮＵ）  What is your age? (NU) |  |
| あなたの最終学歴をお答えください。在学中の方は、現在在籍している学校をお答えください。（ＳＡ）  Please indicate the last level of education attended. If you are currently enrolled, please answer with the level you are currently attending. (SA) | 中学校卒 Middle School 高等学校卒／高等専門学校卒  High School／High School Level Vocational School 短期大学／専門学校卒  Junior College／Vocational School 大学卒 Bachelor’s Degree 大学院修士修了 Master’s Degree 大学院博士修了 Doctoral Degree |
| 主なお仕事内容にあてはまるものを教えてください。（ＳＡ）  Please indicate which occupational industry is the most applicable to your current job. (SA) | 農業，林業 Agriculture and Forestry 建設業 Construction 製造業 Manufacturing 情報通信業 IT and Telecommunications 運輸業，郵便業 Transportation and Postal 卸売業，小売業 Wholesale and Retail Trade 金融業，保険業 Finance and Insurance 不動産業，物品賃貸業 Real Estate and Goods Leasing 学術研究，専門・技術サービス業 Scientific Research, Professional and Technical Services 宿泊業，飲食サービス業 Accommodations and Food Services 生活関連サービス業，娯楽業 Living-Related Personal and Amusement Services 教育，学習支援業 Education and Learning Support 医療，福祉 Medical and Healthcare 複合サービス事業 Compound Services サービス業（他に分類されないもの） Other Services 公務 Public Service 学生 Student 専業主ふ Homemaker その他 Other |
| ２０２０年の世帯年収についてあてはまるものを教えてください。（ＳＡ）  What was your annual household income for 2020? (SA) | １００万円未満 Less than 1 million yen １００～２００万円未満 1 to 2 million yen ２００～３００万円未満 2 to 3 million yen  ３００～４００万円未満 3 to 4 million yen ４００～５００万円未満 4 to 5 million yen ５００～６００万円未満 5 to 6 million yen ６００～７００万円未満 6 to 7 million yen ７００～８００万円未満 7 to 8 million yen ８００～９００万円未満 8 to 9 million yen ９００～１０００万円未満 9 to 10 million yen １０００万円以上 Over 10 million yen |
| Health Related Questions |  |
| 定期予防接種を受けていますか？（ＳＡ） Have you recieved your routine vaccinations? (SA) | 全て受けている Yes  一部受けている Some of them 全て受けていない No わからない Unsure |
| 新型コロナウイルスのワクチンを１回でも接種しましたか？（ＳＡ）  Have you received at least one dose of an approved COVID-19 vaccine? (SA)  (New for 2022 survey) | 市区町村が実施する「集団接種」あるいは「個別接種」で１回目を受けた（接種予約済み含む）Received the first dose of "mass vaccination" or "individual vaccination" administered by the municipality (including those with vaccination reservations)  国や都道府県が実施する「大規模接種」で１回目を受けた（接種予約済み含む）Received the first dose of "mass vaccinations" administered by the national or prefectural government (including those with vaccination reservations)  企業や大学の実施する「職域接種」で１回目を受けた（接種予約済み含む）Received the first dose of "Workplace Vaccinations" administered by companies and universities (including those with vaccination reservations)  受けたいがまだ接種予約ができていない Want to receive the vaccination but have not yet made an appointment  受けるかどうか迷っている Not sure if I will receive the COVID-19 vaccination or not  受けるつもりはない Do not intend to receive the COVID-19 vaccine  関心がない Not interested |
| 既にワクチン接種を受けた方は１回目を受けた当時、受けていない方は今現在、ご自身の周りではどれくらいの方が接種を受けていますでしょうか（いましたでしょうか）。あなたの感覚に最も近いものをお答えください。（ＳＡ）  If you have already been vaccinated, how many people around you were vaccinated at the time you received the first dose, and if not, how many people around you are (or were) vaccinated now? Please answer by your personal perception (SA)  (New for 2022 survey) | ０％  ２５％  ５０％  ７５％  １００％ |
| Psychological Related Questions |  |
| 新型コロナウイルスのワクチンについて、メリットはどのくらい大きいと感じていますか？あなたの認識でお答えください。（ＳＡ）  How significant do you feel the benefits are regarding the vaccine for COVID-19? Please answer based on your perception. (SA) | とても小さい Very insignificant 小さい Insignificant どちらでもない Neutral 大きい Significant とても大きい Very significant |
| 新型コロナウイルスのワクチンについて、デメリットはどのくらい大きいと感じていますか？あなたの認識でお答えください。（ＳＡ）  How significant do you feel the disadvantages are regarding the vaccine for COVID-19? Please answer based on your perception. (SA) | とても小さい Very insignificant 小さい Insignificant どちらでもない Neutral 大きい Significant  とても大きい Very significant |
| 新型コロナウイルスのワクチンの承認について、公的機関を信頼していますか？（ＳＡ）Do you trust public authorities to approve vaccines for novel coronaviruses? (SA) | まったく信頼していない Strongly disagree 信頼していない Disagree どちらでもない Neutral 信頼している Agree 強く信頼している Strongly agree |
| 他の人が新型コロナウイルスのワクチンを接種していたら、自分もワクチン接種をするべきだと思いますか？（ＳＡ） If others have been vaccinated against novel coronavirus, do you think you should be vaccinated too?(SA) | まったくそう思わない Strongly disagree そう思わない Disagree どちらでもない Neutral そう思う Strongly agree 強くそう思う Agree |
| 新型コロナウイルスについて、どの程度不安がありますか？（ＳＡ）  How concerned are you about COVID-19? (SA) | 全然不安はない　I'm not worried at all  漠然とした不安がある　Somewhat concerned  明確に不安を感じている　Anxious  不安に加え恐怖を感じている　Fearful and anxious |
| この１年間に、新型コロナウイルスの流行により、あなたの生活への支障はどの程度ありましたか？（ＳＡ）  During the past year, to what extent has COVID-19 pandemic interfered with your life? (SA) | 全くなかった Not at all  あまりなかったNot much  ある程度あったTo some extent  非常にあったVery much so |
| Information Related Questions |  |
| 新型コロナウイルスに関する情報源として、以下の情報源からの情報をどの程度信頼していますか？それぞれ４段階でお選びください。（ＳＡ）  How much do you trust information about COVID-19 from the following information source? Please select on a scale of 1 to 4 for each. (SA)  <List of information sources> • 新型コロナウイルス感染症対策専門家会議（現分科会）Novel Coronavirus Expert Meeting • 政府 National Government • 都道府県や市町村などの自治体 Local Government • 獣医 Veterinarians • 薬剤師 Pharmacists • 看護師 Nurses • 医師 Doctors  • 歯科医 Dentists • テレビ Television • ラジオ Radio • 新聞 Newspaper • 雑誌 Magazines • 本 Books • 科学者や研究者 Scientists & Researchers • 製薬企業 Pharmaceutical Companies • 友達 Friends • 家族 Family • ＹｏｕＴｕｂｅ／ユーチューブ（ＳＡ）  YouTube • Ｔｗｉｔｔｅｒ／ツイッター Twitter • ＴｉｋＴｏｋ／ティックトック TikTok • 検索エンジン（Google、Yahooなど）  Search Engines • ネットのニュースサイト Online News Sites • ＬＩＮＥ／ライン LINE • Ｉｎｓｔａｇｒａｍ／インスタグラム  Instagram • Ｆａｃｅｂｏｏｋ／フェイスブック  Facebook  • 他の会社または企業  Other Firms or Companies • 医療系情報サイト（Ｍｅｄｉｃａｌ　Ｎｏｔｅ、ＭＥＤＬＥＹ、アピタルなど）  Medical Information Sites • 健康フェア・イベントHealth Fairs & Events • ブログまたは有名人、著名人のウェブペー  ジCelebrity Blogs/Web Pages • 科学文献（論文）Scientific Literature | 全く信用していない Do not trust at all ある程度信頼している Trust to some extent 信頼している Trusting 非常に信頼している Very trusting |
| 新型コロナウイルスのワクチン接種の判断に、以下の情報源から情報はどの程度参考にしましたか？それぞれ４段階でお選びください。  To what extent did you rely on information from the following sources for your COVID-19 vaccine decision? Please select each on a scale of four levels.  <List of information sources>  ※The same as in the upper row | 参考にしていない Do not rely on  どちらかといえば参考にしていない Somewhat do not rely on  どちらかといえば参考にしている Somewhat rely on  参考にしている Rely on |

NU: Numerical answer
SA: Single answer
MA: Multiple answer

*Supplementary Table 2: Grouping and Naming of Variables*

| Variables Prior to Grouping | Post-grouping Variables |
| --- | --- |
| Outcome |  |
| When it becomes possible for children under 12 to receive the COVID-19 vaccine in the future, which of the following applies to you?   - Agree (have children of the relevant age) - Agree (have no children of the relevant age - Disagree (have children of the relevant age) - Disagree (have no children of the relevant age) - Not sure (have children of the relevant age) - Not sure (have no children of the relevant age)   ※"have no children of the relevant age" options were excluded for analysis | - Agree - Disagree - Not sure |
| Sociodemographic Variables |  |
| Which prefecture do you live in? | 6 Regions   - Hokkaido & Tohoku - Kanto - Chubu - Kansai - Chugoku & Shikoku - Kyushu & Okinawa |
| Which prefecture do you live in?  ※Residing in the areas subject to intensive measures for preventing the spread of infection during the survey period | Intensive measures area   - Yes : (Hokkaido, Aomori, Yamagata ,Fukushima,   Ibaraki, Tochigi, Gunma, Saitama, Chiba, Tokyo, Kanagawa, Niigata, Toyama, Ishikawa, Nagano, Gifu, Shizuoka, Aichi, Mie, Kyoto, Osaka, Hyogo, Wakayama, Shimane, Okayama, Hiroshima, Yamaguchi, Kagawa, Kochi, Fukuoka, Saga, Nagasaki, Kumamoto, Oita, Miyazaki, Kagoshima, Okinawa)   - No |
| What is your gender? Please answer by your personal perception.   - Women - Men - Other（※Excluded due to extremely small sample size） | Gender   - Women - Men |
| What is your age? | Age category   - 20-34 - 35-39 - 40-44 - 45- |
| Please indicate the last level of education attended. If you are currently enrolled, please answer with the level you are currently attending. • Middle School • High School/High School Level Vocational  School • Junior College/Vocational School • Bachelor’s Degree • Master’s Degree • Doctoral Degree | Highest educational level • Middle/High school • Junior College/Vocational School • Bachelors/Masters/Doctors Degree |
| Please indicate which occupational industry is the most applicable to your current job. • Agriculture and Forestry • Construction • Manufacturing • IT and Telecommunications • Transportation and Postal • Wholesale and Retail Trade • Finance and Insurance • Real Estate and Goods Leasing • Scientific Research, Professional and Technical Services • Accommodations and Food Services • Living Related Personal and Amusement  Services • Education and Learning Support • Medical and Healthcare • Compound Services • Other Services • Public Service • Student • Homemaker • Other | Occupation • Medical and Healthcare • Manufacturing • Student / Homemaker • Other |
| What was your annual household income for 2020? • Less than 1 million yen • 1-2 million yen • 2-3 million yen • 3-4 million yen • 4-5 million yen • 5-6 million yen • 6-7 million yen • 7-8 million yen • 8-9 million yen • 9-10 million yen • Over 10 million yen | Household income brackets in 2020 • Less than 3 million yen • 3-4 million yen • 4-5 million yen • 5-6 million yen • 6-7 million yen • 7-8 million yen • 8-10 million yen • Over 10 million yen |
| Health-Related Variables |  |
| Have you received your routine vaccinations? | Routine vaccinations |
| Have you received at least one dose of an approved COVID-19 vaccine?   - Received the first dose of "mass vaccination" or "individual vaccination" administered by the municipality (including those with vaccination reservations) - Received the first dose of "mass vaccinations" administered by the national or prefectural government (including those with vaccination reservations) - Received the first dose of "Workplace Vaccinations" administered by companies and universities (including those with vaccination reservations) - Want to receive the vaccination but have not yet made an appointment - Not sure if I will receive the COVID-19 vaccination or not - Do not intend to receive the COVID-19 vaccine   Not interested | COVID-19 vaccination status   - No - Yes or will |
| If you have already been vaccinated, how many people around you were vaccinated at the time you received the first dose, and if not, how many people around you are (or were) vaccinated now? Please answer by your personal perception. | COVID-19 vaccination status of the surroundings |
| Psychological Related Variables |  |
| How significant do you feel the benefits are regarding the vaccine for COVID-19? Please answer based on your perception. • Very Insignificant • Insignificant • Neutral • Significant • Very Significant | Benefits of Covid-19 vaccination • Insignificant • Significant • Neutral |
| How significant do you feel the disadvantages are regarding the vaccine for COVID-19? Please answer based on your perception. • Very Insignificant • Insignificant • Neutral • Significant • Very Significant | Risks of Covid-19 vaccination • Insignificant • Significant • Neutral |
| Do you trust public authorities to approve vaccines for novel coronaviruses? • Strongly Disagree • Disagree • Neutral • Agree • Strongly Agree | Trust in public authorities • Disagree • Agree • Neutral |
| If others have been vaccinated against novel coronavirus, do you think you should be vaccinated too? • Strongly Disagree • Disagree • Neutral • Agree • Strongly Agree | Vaccination Peer Influence • Disagree • Agree • Neutral |
| How concerned are you about COVID-19? | COVID-19 anxiety |
| During the past year, to what extent has COVID-19 pandemic interfered with your life? | Disruption to daily life |
| Information Related Variables |  |
| How much do you trust information about COVID-19 from the following information source?   - Do not trust at all - Trust to some extent - Trusting - Very trusting | Information source   - 1 - 2 - 3 - 4 |
| Consolidation of categories of information sources in “Information source” and the results of Cronbach's alpha and Spearman-Brown test | |
| • Doctors • Nurse • Pharmacist • Veterinarian • Dentist | Trust specialists  (Cronbach's alpha / 0.6429) |
| • Newspaper • Television | Trust mass media  (Spearman-Brown / 0.6888) |
| • Magazines • Books  • Scientific literature (papers)  （※Excluded due to high correlation with other categories） | - |
| • Search engines (Google, Yahoo, etc.) • LINE　 • Facebook • Twitter • Instagram • YouTube • TikTok　 • Medical information sites (Medical Note,  MEDLEY , APITAL, etc.)  • Blogs or web pages of famous and celebrities  • Online news sites | Trust social media and websites  (Cronbach's alpha / 0.6262) |
| • Local governments such as prefectures and  municipalities • Government • Expert Group on Countermeasures to Combat  New Coronavirus Infections | Trust government  (Cronbach's alpha / 07677) |
| • Friends • Family | Trust close people  (Spearman-Brown / 0.7040) |
| • Pharmaceutical companies • Other firms or companies  • Scientists and researchers  （※Excluded due to high correlation with other categories） |  |
| To what extent did you rely on information from the following sources for your COVID-19 vaccine decision? Please select each on a scale of four levels. (select for each information sources)   - Do not rely on - Somewhat do not rely on   ⇒Categorized as “Not utilized”   - Somewhat relied on - Rely on   ⇒Categorized as “utilized”  <List of information sources>  ※The same as in “Information source” row | The number of referenced information sources utilized for COVID-19 |

*Supplementary Table 3: Characteristics of Parents*

|  | **Disagree**  **(n=468, 19.4%)** | **Agree  (n=1049, 43.4%)** | **Not sure  (n=902, 37.3%)** | **Total (n=2,419)** |
| --- | --- | --- | --- | --- |
| **Sociodemographic characteristics** | |  |  |  |
| **6 Regions** | | | | |
| Hokkaido/Tohoku | 49 (20.1/10.5) | 106 (43.4/10.1) | 89 (36.5/9.9) | 244 (100/10.1) |
| Kanto | 149 (17.7/31.8) | 372 (44.1/35.5) | 323 (38.3/35.8) | 844 (100/34.9) |
| Tyubu | 96 (21.1/20.5) | 198 (43.4/18.9) | 162 (35.5/18.0) | 456 (100/18.9) |
| Kansai | 82 (20.6/17.5) | 155 (38.9/14.8) | 162 (40.6/18.0) | 399 (100/16.5) |
| Tyugoku/Shikoku | 42 (18.5/9.0) | 101 (44.5/9.6) | 84 (37.0/9.3) | 227 (100/9.4) |
| Kyushu/Okinawa | 50 (20.1/10.7) | 117 (47.0/11.2) | 82 (32.9/9.1) | 249 (100/10.3) |
| Total | 468 (19.4/100) | 1049 (43.4/100) | 902 (37.2/100) | 2419 (100/100) |
| **Intensive measures area** |  |  |  |  |
| No | 45 (02.0/9.6) | 94 (41.6/9.0) | 87 (38.5/9.7) | 226 (100/9.3) |
| Yes | 423 (19.3/90.4) | 955 (43.6/91.0) | 815 (37.2/90.4) | 2193 (100/90.7) |
| Total | 468 (19.4/100) | 1049 (43.4/100) | 902 (37.3/100) | 2419 (100/100) |
| **Gender** |  |  |  |  |
| Women | 256 (22.6/54.7) | 392 (34.6/37.4) | 485 (42.8/53.8) | 1133 (100/46.8) |
| Men | 212 (16.5/45.3) | 657 (51.1/62.6) | 417 (32.4/46.2) | 1286 (100/53.2) |
| Total | 468 (19.4/100) | 1049 (43.4/100) | 902 (37.3/100) | 2419 (100/100) |
| **Age** | |  |  |  |
| Mean (SD) | 41.5 (11.1) | 46.5 (13.8) | 43.4 (12.9) | 44.4 (13.3) |
| **Age category** |  |  |  |  |
| 20-34 | 115 (23.0/24.6) | 178 (35.6/17.0) | 207 (41.4/23.0) | 500 (100/20.7) |
| 35-39 | 122 (23.3/26.1) | 207 (39.5/19.7) | 195 (37.2/21.6) | 524 (100/21.7) |
| 40-44 | 94 (21.0/20.1) | 185 (41.3/17.6) | 169 (37.7/18.7) | 448 (100/18.5) |
| 45- | 137 (14.5/29.3) | 479 (50.6/45.7) | 331 (35.0/36.7) | 947 (100/39.2) |
| Total | 468 (19.4/100) | 1049 (43.4/100) | 902 (37.3/100) | 2419 (100/100) |
| **Highest educational level** |  |  |  |  |
| Middle/High school | 114 (16.9/24.4) | 286 (42.3/27.3) | 276 (40.8/30.6) | 676 (100/28.0) |
| Junior College / Vocational | 93 (21.2/19.9) | 165 (37.7/15.7) | 180 (41.1/20.0) | 438 (100/18.1) |
| Bachelor's / Master's /Doctoral | 261 (20.0/55.8) | 598 (45.8/57.0) | 446 (34.2/49.5) | 1305 (100/54.0) |
| Total | 468 (19.4/100) | 1049 (43.4/100) | 902 (37.3/100) | 2419 (100/100) |
| **Occupation** |  |  |  |  |
| Medical and Healthcare workers | 41 (19.3/8.8) | 89 (41.8/8.5) | 83 (39.0/9.2) | 213 (100/8.8) |
| Manufacturing | 69 (17.5/14.7) | 193 (49.0/18.4) | 132 (33.5/14.6) | 394 (100/16.3) |
| Student/Homemaker | 128 (24.3/27.4) | 161 (30.6/15.4) | 237 (45. 2/ 16.3) | 526 (100/21.7) |
| Other | 230 (17.9/49.2) | 606 (47.1/57.8) | 450 (35.0/49.9) | 1286 (100/53.2) |
| Total | 468 (19.4/100) | 1049 (43.4/100) | 902 (37.3/100) | 2419 (100/100) |
| **Household income brackets in 2020** | |  |  |  |
| Less than 3 million yen | 65 (17.8/13.9) | 144 (39.5/13.7) | 156 (42.7/17.3) | 365 (100/15.1) |
| 3-4 million yen | 43 (16.0/9.2) | 107 (39.8/10.2) | 119 (44.2/13.2) | 269 (100/11.1) |
| 4-5 million yen | 63 (19.6/13.5) | 141 (43.9/13.4) | 117 (36.5/13.0) | 321 (100/13.3) |
| 5-6 million yen | 72 (20.8/15.4) | 135 (38.9/12.9) | 140 (40.4/15.5) | 347 (100/14.3) |
| 6-7 million yen | 67 (22.9/14.3) | 123 (42.0/11.7) | 103 (35.2/11.4) | 293 (100/12.1) |
| 7-8 million yen | 48 (20.3/10.3) | 112 (47.3/10.7) | 77 (32.5/8.5) | 237 (100/9.8) |
| 8-10 million yen | 50 (16.6/10.7) | 140 (46.5/13.4) | 111 (36.9/12.3) | 301 (100/12.4) |
| More than 10 million yen | 60 (21.0/12.8) | 147 (51.4/14.0) | 79 (27.6/8.8) | 286 (100/11.8) |
| Total | 468 (19.4/100) | 1049 (43.4/100) | 902 (37.3/100) | 2419 (100/100) |
| **Health-related characteristics** | |  |  |  |
| **Routine vaccinations** | |  |  |  |
| Yes | 158 (19.9/33.8) | 364 (45.9/34.7) | 271 (34.2/30.0) | 793 (100/32.8) |
| Some | 76 (17.7/16.2) | 223 (51.9/21.3) | 131 (30.5/14.5) | 430 (100/17.8) |
| No | 160 (20.8/34.2) | 319 (41.5/30.4) | 290 (37.7/32.2) | 769 (100/31.8) |
| Not sure | 74 (17.3/15.8) | 143 (33.5/13.6) | 210 (49.2/23.3) | 427 (100/17.7) |
| Total | 468 (19.4/100) | 1049 (43.4/100) | 902 (37.3/100) | 2419 (100/100) |
| **COVID-19 vaccination status** | |  |  |  |
| No | 172 (51.2/36.8) | 44 (13.1/4.2) | 120 (35.7/13.3) | 336 (100/13.9) |
| Yes or Will | 296 (14.2/63.3) | 1005 (48.3/95.8) | 782 (37.5/86.7) | 2083 (100/86.1) |
| Total | 468 (19.4/100) | 1049 (43.4/100) | 902 (37.3/100) | 2419 (100/100) |
| **COVID-19 vaccination status of the surroundings** | | |  |  |
| Around 0-25% | 98 (23.3/20.9) | 182 (43.3/17.4) | 140 (33.3/15.5) | 420 (100/17.4) |
| Around 50% | 127 (25.6/27.1) | 185 (37.2/17.6) | 185 (37.2/20.5) | 497 (100/21.0) |
| Around 75% | 180 (19.0/39.0) | 399 (42.1/38.0) | 368 (38.9/40.8) | 947 (100/39.2) |
| Around 100% | 63 (11.4/13.5) | 283 (51.0/27.0) | 209 (37.7/23.2) | 555 (100/23.0) |
| Total | 468 (19.4/100) | 1049 (43.4/100) | 902 (37.3/100) | 2419 (100/100) |
| **Psychological characteristics** | |  |  |  |
| **Benefits of COVID-19 vaccination** | |  |  |  |
| Insignificant | 145 (54.7/31.0) | 79 (29.8/7.53) | 41 (15.5/4.6) | 265 (100/11.0) |
| Significant | 114 (7.9/24.4) | 790 (54.8/75.3) | 539 (37.4/59.8) | 1443 (100/60.0) |
| Neutral | 209 (29.4/44.7) | 180 (25.3/17.2) | 322 (45.3/35.7) | 711 (100/29.4) |
| Total | 468 (19.4/100) | 1049 (43.4/100) | 902 (37.3/100) | 2419 (100/100) |
| **Risks of COVID-19 vaccination** | |  |  |  |
| Insignificant | 71 (8.4/15.2) | 538 (64.0/51.3) | 232 (27.6/25.7) | 841 (100/34.8) |
| Significant | 189 (36.3/40.4) | 182 (34.9/17.4) | 150 (28.8/16.6) | 521 (100/21.5) |
| Neutral | 208 (19.7/44.4) | 329 (31.1/31.4) | 520 (49.2/57.7) | 1057 (100/43.7) |
| Total | 468 (19.4/100) | 1049 (43.4/100) | 902 (37.3/100) | 2419 (100/100) |
| **Trust in public authorities** | |  |  |  |
| Disagree | 191 (51.2/40.8) | 93 (24.9/8.9) | 89 (23.9/9.9) | 373 (100/15.4) |
| Agree | 85 (7.5/18.2) | 669 (59.1/63.8) | 378 (33.4/41.9) | 1132 (100/46.8) |
| Neutral | 192 (21.0/41.0) | 287 (31.4/27.4) | 435 (47.6/48.2) | 914 (100/37.8) |
| Total | 468 (19.4/100) | 1049 (43.4/100) | 902 (37.3/100) | 2419 (100/100) |
| **Perceived peer Influence** | |  |  |  |
| Disagree | 180 (55.2/38.5) | 71 (21.8/6.8) | 75 (23.0/8.3) | 326 (100/13.5) |
| Agree | 125 (9.05/26.7) | 788 (57.1/75.1) | 468 (33.9/51.9) | 1381 (100/57.1) |
| Neutral | 163 (22.9/34.8) | 190 (26.7/18.1) | 359 (50.4/39.8) | 712 (100/29.4) |
| Total | 468 (19.4/100) | 1049 (43.4/100) | 902 (37.3/100) | 2419 (100/100) |
| **COVID-19 anxiety** |  |  |  |  |
| I'm not worried at all | 93 (34.2/19.9) | 101 (37.1/9.6) | 78 (28.7/8.7) | 272 (100/11.2) |
| Somewhat concerned | 244 (17.6/52.1) | 600 (43.3/57.2) | 543 (39.2/60.2) | 1387 (100/57.3) |
| Anxious | 101 (17.5/21.6) | 255 (44.2/24.3) | 221 (38.3/24.5) | 577 (100/23.9) |
| Fearful and anxious | 30 (16.4/6.4) | 93 (50.8/8.9) | 60 (32.8/6.7) | 183 (100/7.57) |
| Total | 468 (19.4/100) | 1049 (43.4/100) | 902 (37.3/100) | 2419 (100/100) |
| **Disruption to daily life** | |  |  |  |
| Not at all or not much | 151 (19.6/32.3) | 353 (45.7/33.7) | 268 (34.7/29.7) | 772 (100/31.9) |
| To some extent | 213 (17.8/45.5) | 510 (42.5/48.6) | 477 (39.8/52.9) | 1200 (100/49.6) |
| Yes | 104 (23.3/22.2) | 186 (41.6/17.7) | 157 (35.1/17.4) | 447 (100/18.5) |
| Total | 468 (19.4/100) | 1049 (43.4/100) | 902 (37.3/100) | 2419 (100/100) |
| **Information sources regarding COVID-19** | |  |  |  |
| **Trust specialists** |  |  |  |  |
| Mean [20] | 2.2 (0.6) | 2.5 (0.7) | 2.4 (0.6) | 2.4 (0.6) |
| **Trust mass media** |  |  |  |  |
| Mean [20] | 2.0 (0.7) | 2.4 (0.7) | 2.3 (0.6) | 2.3 (0.7) |
| **Trust SNS websites** |  |  |  |  |
| Mean [20] | 1.8 (0.6) | 1.9 (0.6) | 1.8 (0.6) | 1.8 (0.6) |
| **Trust government** |  |  |  |  |
| Mean [20] | 2.0 (0.7) | 2.5 (0.7) | 2.3 (0.7) | 2.3 (0.7) |
| **Trust close people** |  |  |  |  |
| Mean [20] | 2.1 (0.7) | 2.3 (0.7) | 2.2 (0.6) | 2.2 (0.7) |
| **The number of referenced information sources utilized for COVID-19** | | |  |  |
| Mean [20] | 9.8 (9.2) | 14.5 (9.6) | 13.6 (9.9) | 13.2 (9.8) |

*Supplementary Table 4: Factors Associated with Parental Hesitation on Parental Hesitation on COVID-19 Vaccination for Children*

|  | **Agree** |  | **Not sure** |  |
| --- | --- | --- | --- | --- |
|  | OR (95% conf.interval) | *p*-value | OR (95% conf.interval) | *p*-value |
| **Sociodemographic characteristics** | |  |  |  |
| **6 Regions** |  | | | |
| Hokkaido/Tohoku | 1 (Ref. group) | | 1 (Ref. group) | |
| Kanto | 1.41 (0.85-2.36) | 0.19 | 1.37 (0.83-2.25) | 0.22 |
| Tyubu | 0.98 (0.58-1.67) | 0.94 | 0.95 (0.57-1.60) | 0.86 |
| Kansai | 1.11 (0.64-1.91) | 0.72 | 1.32 (0.78-2.23) | 0.31 |
| Tyugoku/Shikoku | 1.27 (0.69-2.35) | 0.44 | 1.19 (0.66-2.16) | 0.56 |
| Kyushu/Okinawa | 1.19 (0.64-2.19) | 0.59 | 0.95 (0.52-1.72) | 0.86 |
| **Intensive measures area** |  |  |  |  |
| No | 1 (Ref. group) |  | 1 (Ref. group) |  |
| Yes | 1.18 (0.72-1.94) | 0.52 | 1.08 (0.67-1.73) | 0.76 |
| **Gender** |  |  |  |  |
| Women | 1 (Ref. group) |  | 1 (Ref. group) |  |
| Men | 1.55 (1.12-2.17) | 0.01 | 1.09 (0.79-1.50) | 0.60 |
| **Age category** |  |  |  |  |
| 20-34 | 1 (Ref. group) |  |  |  |
| 35-39 | 0.81 (0.55-1.21) | 0.31 | 0.78 (0.54-1.13) | 0.19 |
| 40-44 | 0.91 (0.59-1.39) | 0.66 | 0.97 (0.65-1.45) | 0.87 |
| 45- | 1.17 (0.80-1.72) | 0.41 | 1.10 (0.76-1.59) | 0.61 |
| **Highest educational level** | | | | |
| Middle / High school | 1 (Ref. group) | | 1 (Ref. group) | |
| Junior College / vocational | 0.61 (0.40- 0.94) | 0.02 | 0.62 (0.42-0.93) | 0.02 |
| Bachelor's / Master's / Doctoral | 0.60 (0.42-0.86) | 0.01 | 0.59 (0.42-0.82) | <0.01 |
| **Occupation** |  | | | |
| Medical and Healthcare | 1 (Ref. group) | | 1 (Ref. group) | |
| Manufacturing | 1.09 (0.61- 1.96) | 0.77 | 0.88 (0.50-1.56) | 0.66 |
| Student / Homemaker | 0.50 (0.29-0.86) | 0.01 | 0.73 (0.44-1.21) | 0.22 |
| Other | 0.94 (0.57--1.55) | 0.82 | 0.87 (0.54-1.41) | 0.57 |
| **Household income brackets in 2020** | |  | |  |
| Less than 3 million yen | 1 (Ref. group) |  | 1 (Ref. group) |  |
| 3-4 million yen | 1.22 (0.70-2.13) | 0.49 | 1.30 (0.77-2.17) | 0.33 |
| 4-5 million yen | 1.16 (0.69-1.94) | 0.57 | 1.00 (0.61-1.65) | 0.99 |
| 5-6 million yen | 0.87 (0.52-1.44) | 0.58 | 0.92 (0.57-1.47) | 0.72 |
| 6-7 million yen | 1.21 (0.72-2.04) | 0.47 | 0.91 (0.55-1.49) | 0.70 |
| 7-8 million yen | 1.28 (0.73-2.26) | 0.39 | 0.90 (0.52-1.56) | 0.71 |
| 8-10 million yen | 1.44 (0.83-2.48) | 0.19 | 1.23 (0.73-2.08) | 0.44 |
| Over 10 million yen | 1.00 (0.58-1.72) | 1.00 | 0.61 (0.36-1.05) | 0.07 |
| **Health related factors** | | | | |
| **Routine vaccinations** |  | | | |
| Yes | 0.75 (0.53-1.06) | 0.11 | 0.69 (0.49-0.98) | 0.04 |
| Some of them | 1.08 (0.72-1.62) | 0.72 | 0.78 (0.52-1.17) | 0.22 |
| No | 1 (Ref. group) | | 1 (Ref. group) | |
| Not sure | 0.89 (0.59-1.36) | 0.60 | 1.26 (0.85-1.86) | 0.25 |
| **COVID-19 vaccination status** | | | | |
| No | 1 (Ref. group) | | 1 (Ref. group) | |
| Yes or Will | 3.48 (2.25-5.38) | <0.01 | 1.29 (0.91-1.84) | 0.16 |
| **COVID-19 vaccination status of the surroundings** | | | | |
| Around 0-25% | 1 (Ref. group) | | 1 (Ref. group) | |
| Around 50% | 1.05 (0.69-1.59) | 0.82 | 1.17 (0.78-1.74) | 0.45 |
| Around 75% | 1.13 (0.78-1.65) | 0.52 | 1.30 (0.90-1.88) | 0.16 |
| Around 100% | 1.84 (1.18-2.88) | 0.01 | 2.00 (1.29-3.10) | <0.01 |
| **Psychological factors** | | | | |
| **Benefits of COVID-19 vaccination** | | | | |
| Insignificant | 1 (Ref. group) | | 1 (Ref. group) | |
| Significant | 2.05 (1.27-3.30) | <0.01 | 5.08 (3.09-8.30) | <0.01 |
| Neutral | 0.73 (0.45-1.17) | 0.19 | 1.79 (1.11-2.89) | 0.02 |
| **Risks of COVID-19 vaccination** | | | | |
| Insignificant | 1 (Ref. group) | | 1 (Ref. group) | |
| Significant | 0.29 (0.19-0.43) | <0.01 | 0.48 (0.32-0.71) | <0.01 |
| Neutral | 0.48 (0.33-0.71) | <0.01 | 1.14 (0.77-1.68) | 0.52 |
| **Trust in public authorities** | | | | |
| Disagree | 1 (Ref. group) | | 1 (Ref. group) | |
| Agree | 3.23 (2.04-5.11) | <0.01 | 2.78 (1.79-4.32) | <0.01 |
| Neutral | 1.65 (1.05-2.59) | 0.03 | 2.06 (1.43-2.98) | <0.01 |
| **Perceived peer influence** | | | | |
| Disagree | 1 (Ref. group) | | 1 (Ref. group) | |
| Agree | 3.23 (2.04-5.11) | <0.01 | 1.96 (1.26-3.03) | <0.01 |
| Neutral | 1.65 (1.05-2.59) | 0.03 | 2.13 (1.41-3.19) | <0.01 |
| **COVID-19 anxiety** |  | | | |
| I'm not worried at all | 1 (Ref. group) | | 1 (Ref. group) | |
| Somewhat concerned | 1.36 (0.88-2.10) | 0.16 | 1.63 (1.07-2.48) | 0.02 |
| Anxious | 1.30 (0.79-2.15) | 0.31 | 1.55 (0.95-2.52) | 0.08 |
| Fearful and anxious | 3.17 (1.61-6.25) | 0.00 | 2.25 (1.15-4.37) | 0.02 |
| **Disruption to daily life** | | | | |
| Not at all or not much | 1 (Ref. group) | | 1 (Ref. group) | |
| To some extent | 0.66 (0.47-0.91) | 0.01 | 0.82 (0.60-1.13) | 0.23 |
| Yes | 0.45 (0.29-0.68) | <0.01 | 0.59 (0.39-0.88) | 0.01 |
| **Information related factors** | | | | |
| **Trust in information sources** | | | | |
| Trust specialists | 1.27 (0.95-1.70) | 0.11 | 1.06 (0.80-1.41) | 0.70 |
| Trust mass media | 1.25 (0.94-1.68) | 0.13 | 1.24 (0.92-1.65) | 0.15 |
| Trust SNS websites | 0.86 (0.62-1.18) | 0.35 | 0.60 (0.44-0.83) | <0.01 |
| Trust government | 1.04 (0.78-1.39) | 0.79 | 1.03 (0.78-1.38) | 0.82 |
| Trust close people | 0.86 (0.67-1.11) | 0.26 | 0.94 (0.74-1.21) | 0.65 |
| **The number of information sources utilized for COVID-19** | | | | |
|  | 1.02 (1.01-1.04) | 0.01 | 1.03 (1.01-1.04) | <0.01 |

*Supplementary Table 5: Differences between non-respondents and respondents in the second survey*

|  | **Non-respondents (n=10826)** | **Respondents (n=19174)** | **p-value** |
| --- | --- | --- | --- |
| **Gender** |  |  |  |
| women | 6160 (56.9) | 9430 (49.2) | <0.01 |
| men | 4666 (43.1) | 9744 (50.8) |  |
| **Age** |  |  |  |
| Mean (SD) | 48.1 (0.17) | 54.7 (0.11) | <0.01 |
|  |  |  |  |
| **Highest educational level** | |  |  |
| Middle / High school | 4023 (37.2) | 6966 (36.3) | <0.01 |
| Junior College / vocational | 2189 (20.2) | 3642 (19.0) |  |
| Bachelor's / Master's / Doctoral | 4614 (42.6) | 8566 (44.7) |  |
| **Occupation** |  |  |  |
| Medical and Healthcare | 746 (6.9) | 1139 (5.9) | <0.01 |
| Manufacturing | 1092 (10.1) | 1973 (10.3) |  |
| Student / Homemaker | 2832 (26.2) | 4514 (23.5) |  |
| Other | 6156 (56.9) | 11548 (60.2) |  |
| **Household income brackets in 2020** | |  |  |
| Less than 3 million yen | 3466 (32.0) | 5495 (28.7) | <0.01 |
| 3-4 million yen | 1613 (14.9) | 3022 (15.8) |  |
| 4-5 million yen | 1341 (12.4) | 2508 (13.1) |  |
| 5-6 million yen | 1089 (10.1) | 1929 (10.1) |  |
| 6-7 million yen | 782 (7.2) | 1485 (7.7) |  |
| 7-8 million yen | 689 (6.4) | 1287 (6.7) |  |
| 8-10 million yen | 923 (8.5) | 1622 (8.5) |  |
| Over 10 million yen | 923 (8.5) | 1826 (9.5) |  |

*＊Values in parentheses represent column percentages of each category*
